# QuEChERS-Based LC-MS/MS and HRMS Methods for PFAS Determination in Food: A Systematic Review

**DOI:** 10.3390/foods15111872

**Published:** 2026-05-25

**Authors:** Francesco Giuseppe Galluzzo, Gaetano Cammilleri, Licia Pantano, Vittorio Calabrese, Maria Drussilla Buscemi, Elisa Maria Domenica Messina, Calogero Alfano, Dario Bonomo, Andrea Pulvirenti, Andrea Macaluso, Vincenzo Ferrantelli, Gianluigi Maria Lo Dico

**Affiliations:** 1 Istituto Zooprofilattico Sperimentale della Sicilia “A. Mirri”, Via Gino Marinuzzi n. 3, 90129 Palermo, Italy; francesco.galluzzo@izssicilia.it (F.G.G.); gaetano.cammilleri@izssicilia.it (G.C.); licia.pantano@izssicilia.it (L.P.); drussilla.buscemi@izssicilia.it (M.D.B.); calogero.alfano@izssicilia.it (C.A.); dariobonomo.db@gmail.com (D.B.); andrea.macaluso@izssicilia.it (A.M.); vincenzo.ferrantelli@izssicilia.it (V.F.); gianluigi.lodico@izssicilia.it (G.M.L.D.); 2 Dipartimento di Scienze Biomediche e Biotecnologiche, Università Degli Studi di Catania, 95123 Catania, Italy; calabres@unict.it; 3 Dipartimento Scienze della Vita, Università Degli Studi di Modena e Reggio Emilia, 41125 Modena, Italy; andrea.pulvirenti@unimore.it

**Keywords:** food monitoring, method validation, European regulation

## Abstract

Per- and polyfluoroalkyl substances (PFAS) are persistent contaminants that require very strict performance criteria from the methods that want to analyze them in food for research or regulatory purposes. This systematic literature review tried to evaluate Quick, Easy, Cheap, Effective, Rugged, Safe (QuEChERS) extraction methodologies coupled with liquid chromatography-tandem mass spectrometry (LC-MS/MS) and high-resolution mass spectrometry (HRMS) for PFAS determination in food. Peer-reviewed articles (2010–2025) were eligible if they analyzed PFAS in food matrices using QuEChERS extraction protocols with LC-MS/MS or HRMS and reported performance and/or validation data. Scopus, WoS and Google Scholar were searched up to 18 December 2025. Due to heterogeneity in matrices, PFAS panels and reported validation metrics, no meta-analysis was performed, and the results were synthesized narratively. Twenty-four studies met the inclusion criteria. Most methods used acidified acetonitrile (ACN)-based QuEChERS workflows and achieved limits of quantification (LOQ) reported to be compatible with EU Regulation 2023/915 and Commission Implementing Regulation 2022/1428. Analytical scope expanded from 9 to 15 legacy PFAS to >40 analytes. Short-chain PFAS analyses in vegetable matrices and methods from developing countries are underrepresented. QuEChERS-based LC-MS/MS and HRMS methods support regulatory PFAS monitoring and PFAS research. The main limitation of this review is the heterogeneity of included studies and the absence of formal meta-analysis.

## 1. Introduction

PFAS, designated “forever chemicals”, represent critical environmental contaminants due to exceptional persistence and resistance to degradation [1,2,3,4,5]. One of the causes of these properties is the carbon–fluorine bond (Cδ^+^-Fδ^−^), which is one of the strongest in organic chemistry [6]. PFAS have been extensively used since the 1950s in industries (e.g., non-stick cookware, food packaging, and firefighting foams) [7]. Major producers have been reported to conceal health hazard evidence for decades despite PFAS toxicological effects such as liver damage, decreased fertility, and immune dysfunction [8,9,10,11,12,13,14]. The Stockholm Convention (2001/2004) was one of the first legal conventions for global PFAS regulation. The ubiquitous presence of PFAS in the food chain represents a critical food safety concern [15]. Contamination pathways include direct application in food-contact materials, environmental contamination of agricultural systems, and bioaccumulation in livestock and aquatic organisms [3,16,17,18,19,20,21]. PFAS have been detected and reported over the regulatory limits in seafood, meat, dairy, eggs, fruits, vegetables, and processed foods, often exceeding regulatory limits [22,23,24,25,26]. The IARC classified PFOA as carcinogenic to humans (Group 1), and PFOS as possibly carcinogenic (Group 2B), and the EFSA established a tolerable weekly intake of 4.4 ng/kg body weight for four priority PFAS (PFOA, PFOS, PFNA, PFHxS) [27,28,29]. In Europe, Commission Regulation (EU) 2022/2388 (confirmed by Regulation 2023/915) set maximum levels for these four PFAS in specific foodstuffs [30].

The EURL provided harmonized analytical performance guidance in 2022 [30,31,32,33,34]. LC-MS/MS represents the gold standard for quantitative PFAS analysis for regulatory purposes [35]. HRMS (e.g., Orbitrap, TOF) can do non-target screening (NTS) and unknown compound identification, expanding analytical scope beyond regulatory purposes [36,37]. Other analytical techniques have been successfully adopted to overcome the challenges associated with PFAS analysis. The orthogonal separation mechanism of 2D-LC has been used to resolve related homologues and isomeric structures that can remain unresolved in one-dimensional LC [38]. IM-MS enhances spectral quality in complex matrices by introducing an additional gas-phase separation step [39]. Regardless of the analytical method, isotopically labeled internal standards are essential for accurate quantification and for compensating matrix effects and analyte losses [40,41,42,43,44,45,46].

QuEChERS methodology, originally developed for pesticides, has been applied for the analysis of a wide range of analytes with different polarities (pesticides, mycotoxins, and antibiotics) in different matrices, and it is not surprising that several authors tried to transfer this extraction technique to PFAS determination [47,48,49,50,51,52,53,54,55,56]. The main drawback of QuEChERS is that its versatility can result in losses of analytes when conditions are adjusted to maximize the recovery of others [57].

New regulations setting MLs for PFAS in foodstuffs pushed researchers to validate new methods that meet performance criteria [32]. At the same time, some countries, such as France, are progressively restricting the use of PFAS in industries [58]. In the USA, federal methods and regulations establish regulatory frameworks that cover not only PFAS in food and beverages but also PFAS in food-contact and packaging materials [59]. China is continually introducing new regulations aimed at preventing, monitoring and controlling PFAS contamination in the environment and in food [60].

This systematic literature review with qualitative methodological appraisal examines LC-MS/MS and HRMS approaches coupled with QuEChERS extraction protocols for PFAS determination in food matrices. There are other systematic reviews that report different aspects of PFAS (e.g., toxicology, NTS), [37,61,62,63,64,65,66]. However, as far as we know, a validation-oriented synthesis specifically focused on QuEChERS-LC-MS/MS and HRMS applications in food matrices, with a comparison against current EU regulatory criteria, has not previously been conducted. In using an EU benchmark, we tried to establish a reference point for method performance while also recognizing that this perspective has several limitations when extrapolated to non-European regulatory contexts.

## 2. Methods

### 2.1. Objective

The objective of this systematic review was to synthesize QuEChERS-based LC-MS/MS and HRMS methods for PFAS determination in food matrices and to benchmark their validation performance against current EU analytical performance criteria for PFAS in food. This systematic literature review with qualitative methodological appraisal was conducted following Preferred Reporting Items for Systematic Reviews and Meta-Analyses (PRISMA) guidelines (Figure S1, Table S1) [67]. The review was conducted according to an internal protocol that was not registered. Meta-analyses were not performed because of substantial heterogeneity in PFAS panels, food matrices, and reported validation metrics.

### 2.2. Information Sources and Search Strategy, Eligibility Criteria, Study Selection, and Data Collection

The search engines used for the manuscript selection were: Scopus, Web of Science (WOS), and Google Scholar. Search strings used were: (“PFAS” OR “polyfluoroalkyl substances” OR “perfluoro” OR “polyfluoro”) AND (“LC-MS” OR “HRMS” OR “mass spectrometry” OR “LC-MS/MS” OR “UPLC-MS” OR “Orbitrap” OR “Q-TOF”) AND (“QuEChERS” OR “salting-out” OR “dispersive SPE” OR dSPE) AND (“food” OR “dietary” OR “seafood” OR “meat” OR “dairy” OR “eggs” OR “fruits” OR “vegetables” OR “agricultural”). The research was performed on 18 December 2025 for all the databases. For Google Scholar, the first 100 records sorted by relevance were screened. Only manuscripts from 2010 to 2025 were considered for the review. The export from WOS gave 111 results, the export from Google Scholar gave 163 results, and the export from Scopus resulted in 27 documents. Articles from each search engine were combined, and duplicates (n = 45) were identified and removed using reference management software (Zotero 9) based on title, authors, year, and DOI, followed by manual verification. Two independent reviewers screened titles/abstracts and full texts against the eligibility criteria (Table S2); a third reviewer resolved any disagreements. Studies were eligible if they reported sufficient methodological detail to assess extraction, clean-up, and validation performance, and provided data that could be used to evaluate or discuss compliance with EU Regulation 2023/915 and/or Implementing Regulation 2022/1428. We excluded reviews, conference abstracts, books, theses, non-peer-reviewed material, studies outside the 2010–2025 time window, non-food or non-dietary matrices (e.g., serum, environmental or drinking water without a food context), multiclass methods without PFAS-specific performance data, methods lacking any QuEChERS/salting-out/d-SPE step, no MS studies, no English articles, and studies with insufficient methodological detail or no relevance to regulatory contaminant limits.

**Figure 1:**
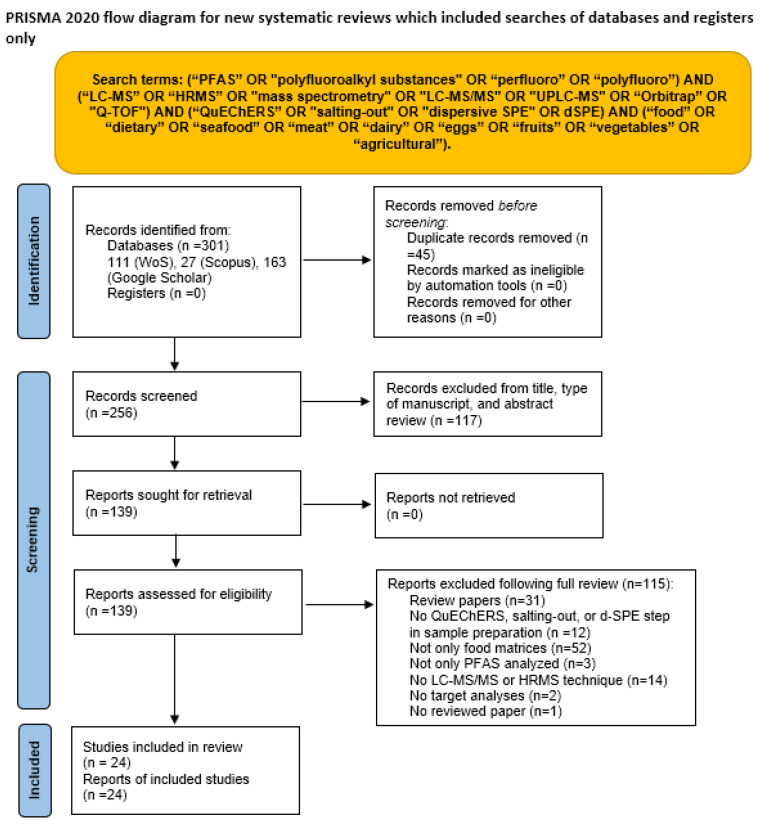
Selection and exclusion processes for the literature review, The flow diagram is adapted from Page, M.J. et al. [67].

No automation tools were used for screening. After removal of duplicates (Figure 1), 256 records were screened by title, document type, and abstract, and 117 were excluded as not relevant. The remaining 139 reports were retrieved and assessed for eligibility. Of these, 115 full-text articles were excluded for the following reasons: review papers (n = 31); no QuEChERS, salting-out, or d-SPE step in sample preparation (n = 12); non-food or non-dietary matrices only (e.g., environmental water) (n = 52); methods without PFAS-specific validation data (n = 3); no LC-MS/MS or HRMS technique (n = 14); no targeted PFAS analysis (n = 2); and not a peer-reviewed article (n = 1). This resulted in a total of 24 articles.

### 2.3. Synthesis Methods

Sources were reviewed considering the matrices analyzed, the extraction procedure, the dSPE utilized for extraction and clean-up, the chromatography and spectrometry parts, the validation procedure, and the compliance with EU Regulation 2023/915 and EU Regulation 2022/1428. Data were systematically collected and organized across four main thematic areas (Table S4): (1) sample matrices and extraction techniques: animal-derived tissues (e.g., fish muscle, poultry muscle), plant-based matrices (coffee, rice, vegetables, fruits), food categories (infant formula, milk, water, edible oils). For each matrix, extraction techniques and physical sample preparation operations employed were considered (e.g., extraction solvents, sample treatment, and separation steps; (2) dSPE characteristics: salt used (e.g., sodium acetate, sodium chloride, magnesium sulfate), mineral phases (magnetite-silica, zirconium dioxide), sorbent materials (PSA, GCB, C18-bonded silica), specific quantities (g or mg) and combination of these reagents; (3) instrumental analyses and quality assurance/quality control (QC): analytical instrumentation (high performance liquid chromatography HPLC column, mobile phase), ionization techniques (ESI, APCI), MS types (triple quadrupole QqQ, Q-TOF, HRMS), quality control procedures (e.g., laboratory blanks, field blanks, procedural blanks, quantitative recovery standards, internal standards, standard reference material SRM); (4) compliance with European regulatory frameworks: EU Regulation 2023/915 MLs, EU Regulation 2022/1428 Table 5 for analytical method performance requirements (selectivity, RSD ≤ 20%, trueness 100 ± 20%, LOQ ≤ maximum level), EU Recommendation 2022/1431 for LOQ in matrices not regulated by EU Regulation 2023/915. If only unregulated matrices by EU Regulation 2023/915 were analyzed, the EU Recommendation 2022/1431 was considered instead for LOQ, even if a recommendation is not mandatory.

Where compliance with EU legislation was not explicitly stated by the authors, the method was classified as “Apparent compliant” if its performance characteristics would meet the criteria in Table 5 of Implementing Regulation (EU) 2022/1428 (applicability regarding foods specified in Regulation (EC) No 1881/2006, selectivity, RSD ≤ 20%, trueness (100 ± 20%, LOQs ≤ MLs, evaluated per analyte) and MLs of EU Regulation 2023/915 [30,68].

“Explicitly claimed compliance” classification was applied when authors stated their method met regulatory criteria. If neither of the two criteria was met, methods were classified as “non-compliant”. The total number of PFAS detected and PFAS classes reported was collected. When several optimization steps were presented, data from the final validated configuration were considered. All extracted data have been tabulated and are reported in Supplementary Information (Tables S4–S7). Data extracted were tabulated and summarized on Excel (Microsoft Office LTSC Standard 2021, v. 2108) in .csv format. Graphs were prepared with R software (4.1.2) (R Core Team).

### 2.4. Risk of Bias (RoB)

A methodological quality and RoB checklist was developed for analytical methods and applied to all included studies. This checklist covered three domains: (A) reporting and transparency, (B) validation criteria and optimization, and (C) quality control and external testing with predefined signaling questions and domain-level ratings (low risk, some concerns, high risk). The overall rating among the three domains was evaluated by the most frequent classification among the three domains. Domain-level scores and overall RoB judgements for each method are reported in Tables S3 and S4 and were used to inform the interpretation of the findings. Potential methodological limitations of individual methods (e.g., low recoveries, matrix effects, limited QC information) were considered qualitatively in the narrative synthesis and are discussed in the limitations section, consistent with similar methodological reviews [69].

## 3. Results and Discussions

### 3.1. Bias Assessment

The results of the RoB of the 24 sources (Tables S3 and S4) reporting for domain A (reporting and transparency, low risks n = 21, some concern n = 3, high concern n = 0) described in a reproducible way a validation design, provided raw data, chromatograms, and analytical parameters (precision, LOQ, accuracy). Domain B (validation criteria and optimization, low risks n = 21, some concern n = 3, high concern n = 0) was usually established a priori and met for the majority of target PFAS, and optimization steps (extraction conditions, dSPE sorbents, matrix-effect control) were documented sufficiently. Domain C (QC and external testing, low risks n = 20, some concern n = 4, high concern n = 0) was at a higher RoB because, despite sources describing blank strategies and applying the method to real food samples, a minority reported participation in interlaboratory studies, use of SRM, or other forms of external verification. However, SRMs, as described in some sources, are not always for matrices (especially fruits and vegetables) [70,71,72].

Most methods combine different PFAS analyses (20–40) in different matrices and achieve recoveries and RSD according to these necessities. In addition, recovery differences are strongly influenced by isotope dilution internal standards, and methods that apply a strict isotope dilution approach could report lower, but more accurate, recoveries. Each method has been optimized for PFAS panel size, matrix size, objectives (regulatory/research objectives), different regulatory limits, study design (QC, QA), instruments, and necessities. For these reasons, we strongly suggest that readers consult our Supplementary Materials, all the sources, and their Supplementary Materials.

### 3.2. Geographical Distribution

The 24 reviewed manuscripts originated from 11 countries, with almost all the sources from developed nations in the Northern Hemisphere (>95% from North America, Europe, and Asia-Pacific). The United States researchers contributed to 20.8% of the manuscripts (n = 5), some of whom were FDA employees who analyzed a wide range of matrices (e.g., cereals, meat, and seafood) [57,71,72,73,74]. Almost all the authors from Asia were from China, and they have contributed equally to the USA (20.8%, five manuscripts), analyzing PFAS in cereals, meat, and dairy [53,75,76,77,78]. Authors from the Republic of Korea investigated the presence of PFAS in rice and coffee in two separate manuscripts (8.3%), while researchers from Australia wrote a single manuscript about PFAS in vegetables [47,79,80]. Collectively, researchers from the Asia-Pacific region accounted for 37.5% of the global output, with authors based in China and the USA together represented 41.6% of the total origin of the manuscripts.

Authors from Europe were the most represented; they contributed 45.8% (10 manuscripts) from six countries. Authors from Italy contributed 12.5% (three manuscripts) and developed methods for the analysis of PFAS in animal matrices and fruits using UHPLC-HRMS [48,70,81]. Two studies conducted in Poland analyzed PFAS in high-protein foods and edible oils [82,83]. Scottish researcher (two manuscripts) analyzed PFAS in milk and processed foods [84,85]. Other European countries represented were France, Belgium, and Switzerland, each of which contributed with one manuscript [15,81,86]. A single manuscript was reported from South Africa, where authors validated a method that allows the analysis of PFAS for milk and infant formula [87]. This represents a fundamental limitation in monitoring the presence of PFAS globally, especially in African countries [88,89,90]. The geographical distribution of references is heavily skewed towards developed countries due to economic cost and regulatory pressure. No references were found from Latin American or Southeast Asian countries. This pattern could reflect not only differences in regulatory systems, but also unequal access to instruments (e.g., LC-MS/MS, HRMS) and funding that is more available in high-income regions [88,91]. Europe, China, and the USA engage in significant food trade, and each has its own regulations about PFAS in food that could influence the analytical focus in these regions. The acceleration of European method publications between 2023 and 2025 could be correlated with the implementation of EU Regulation 2023/915 MLs and EURL-POPs reference laboratory harmonization guidance, demonstrating that regulatory enforcement drives analytical method development and validation cycles [51,92,93,94]. Therefore, the geographical distribution of references does not primarily reflect PFAS exposure but rather where economic resources and regulatory pressure are sufficient to increase PFAS food-monitoring and method-validation activities.

### 3.3. Sample Matrices and Extraction Procedures

#### 3.3.1. Sample Matrices Analyzed

PFAS determination in food requires sample preparation protocols that can extract a wide range of analytes while minimizing matrix interferences [71,95,96]. One of the most complex analytical challenges in determining PFAS in food is the need to cover a wide range of PFAS in diverse food products. Regarding PFAS, they can have different hydrophilic/lipophilic properties; in fact, they can span from short-chain (C4) to long-chain (C18) PFAS. For foods, nutritional properties and physical state can influence PFAS extraction and PFAS–matrix interaction. Despite this challenge, QuEChERS extraction and clean-up steps have been used successfully for routine analysis of PFAS in multi-matrix workflows, including products of both animal and plant origin [97,98,99,100,101,102].

**Figure 2:**
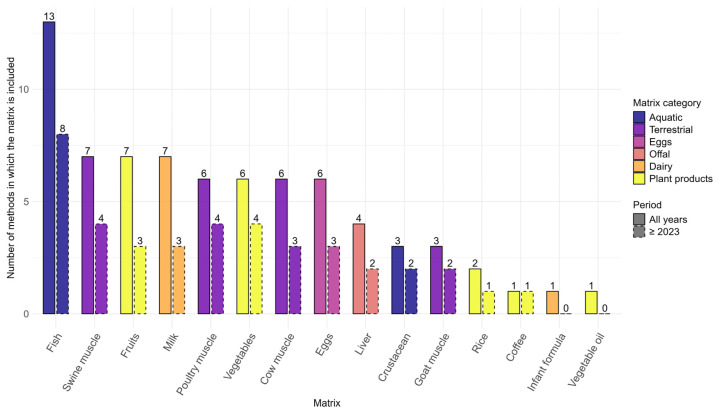
Number of QuEChERS-based PFAS methods in which each food matrix is included. For every matrix, bars represent all identified methods (solid line) and those published from 2023 onwards (dashed line).

Foods of animal origin analyzed in the literature (Figure 2) for PFAS with the QuEChERS extraction included animal muscle, eggs, dairy products (milk, cheese, and yogurt), and offal (liver) (Table S3). Other matrices analyzed were fruits, rice, coffee, oil, and infant formula. Food matrices are not equally tested, and some of them are more analyzed for regulatory priorities and/or toxicological relevance [74]. Fish muscle is the most common matrix analyzed. This could be correlated to the fact that fish have a unique position in aquatic food webs and are linked to human dietary exposure [86,103,104]. In fact, fish can bioaccumulate PFAS (particularly PFOA and PFOS) in their tissues, especially in the liver and muscle, in higher concentrations than surrounding water or sediment [103,105,106]. The same pattern is observed in other animals (cow, goat, swine, poultry), which can accumulate PFAS in their meat and organs [107,108]. Plant product matrices are less frequently analyzed for several reasons. They generally exhibit lower PFAS concentrations and a less diverse profile of PFAS found compared to animal tissues [74].

Plants can take up PFAS from soil and water but generally lack complex bioaccumulation mechanisms found in higher trophic-level animals [109]. However, there is increasing evidence that these matrices can contain PFAS. Surveys in Australia reported that vegetables and fruits can be a reservoir of short-chain PFAS (C4-C7 PFCAs); PFBA was detected in over two-thirds of contaminated samples [80]. Furthermore, long-chain PFAS can bind to plant tissues (e.g., dill, garlic) that contain protein or can deposit on the surface of fruits in addition to root uptake [18]. Another factor that could be correlated with the presence of PFAS in plant-based foods is the packaging. In fact, packaging can be a source of emerging precursors that migrate under specific thermodynamic stresses. Hwang et al. (2025) reported that high pressure and high temperature (61 °C) of espresso machines significantly enhance the migration of 8:2 FTS and PFOA from polypropylene (PP) capsules compared to manual paper-filter brewing [79]. Similarly, boiling instant rice in its original PP packaging resulted in higher PFAS transfer compared to microwave heating [47]. Another reason why these matrices can contain PFAS is that supermarket vegetable bags can be a source of PFAS, especially PFOS, which was found in almost all tested bags [80]. Then, unlike animal tissues, plant matrices contain a broader and more heterogeneous co-extractives compounds (e.g., phenolic compounds and photosynthetic pigments) and are often considered as “matrix-rich and difficult to process”. Some studies adapted QuEChERS workflows originally developed for soil or animal biota to vegetables [72,110]. Furthermore, plant product matrices have heterogeneous PFAS translocation, with short-chain PFAS accumulating in aerial tissues and long-chain PFAS retained in roots [72,111]. These factors complicate the extraction and quantification of PFAS even when using QuEChERS protocols that were originally intended for the extraction of pesticides in fruits and vegetables [70]. Modifications in extraction solvents and clean-up sorbents are required to address the unique chemical compositions of different food groups [70,74]. Matrices such as fish, meat, and milk are characterized by high-protein and lipid contents that can retain long-chain PFAS [53,70,74]. In milk, proteins with binding properties can bind to long-chain PFAS, reducing extraction efficiency [53,70,74]. Lipids and fatty acids are frequently coextracted with PFAS and can reduce the instrumental signal (ion suppression) or coelute with them if not properly removed [53,70,74]. However, animal matrices have been validated in different protocols (EPA/FDA) due to the regulatory pressure, and they are more standardized. To date, there is a scarcity of methods specifically for plant matrices and a lack of comprehensive optimization and validation for PFAS across the wide variety of plant species compared to the work done for animal tissues [110]. Many of the reviewed methods were developed before the current European legislation entered into force. Therefore, further methods are still required to ensure compliance with EU requirements, plant-based matrices remain underrepresented and often rely on adapted protocols that do not fully address their complex co-extractive profiles or the distinct distribution of short- versus long-chain PFAS.

#### 3.3.2. QuEChERS Protocols: General Considerations, Strengths and Challenges

Most methods used ACN-based QuEChERS extractions with EN-type salt mixtures (NaCl, MgSO_4_, and acetate/citrate buffers). ACN is the primary solvent used for the extraction of PFAS (95% of reviewed QuEChERS methods) because it precipitates proteins, solubilizes both short and long-chain PFAS, and is compatible with the salting-out precipitation. Mild acidification with FA, AA, or HCl at 0.1–1.5% (*v*/*v*) improves recoveries by reducing PFAS–matrix interactions; hydrophobic or electrostatic interactions can be disrupted by acid hydrolysis and promoting migration of partially protonated PFAS into the organic phase [70,77,78,83,112]. However, higher acidification (e.g., 2% of FA) can lead to lower recoveries, and a percentage of 0.1–1% is considered a good compromise [73,84]. This could be useful for lowering the recovery rates as a deliberate strategy to stay within the range of ±20% [53]. MeOH, often in combination with KOH at 0.01 M concentration, represents an alternative approach for protein-rich matrices because the alkaline conditions facilitate protein solubilization and can improve the recoveries for certain PFAS [51,73,81,113,114]; ACN provides broader applicability [101,115,116,117]. Mixtures of water and organic solvents (typically ACN or MeOH) in ratios ranging from 1:1 to 1:5 (*v*/*v*) have been used for improving extraction efficiency for PFAS [73,81]. Jeannot et al. reported that an ACN:H_2_O mixture with 1% FA achieved optimal signal intensity and signal-to-noise ratios for 28 target PFAS, outperforming other solvents [81]. The sample/volume ratio depends on the hydration of the sample and the method scope.

The first centrifugation, performed after the salting-out extraction step, separates the ACN phase from the aqueous phase and pellets precipitated proteins and salts. The second centrifugation, carried out after the d-SPE clean-up, separates the pellets from the sorbent materials [118,119,120]. Most methods reviewed have one or more steps of centrifugation that can initially separate the crude extract and then remove any residual particulates before instrumental injection (Table S4). Recent advancements have introduced integrated QuEChERS methods that eliminate the need for centrifugation entirely [53,84]. Magnetic separation reduces total sample preparation time; an external magnetic field applied in a range of 3 to 30 s can achieve solid–liquid separation much faster than traditional centrifugation while maintaining equivalent high recoveries [53,84]. Improper homogenization is a major source of imprecision [116,121]. For liquid samples, vortex mixing can be used instead [87].

The most common QuEChERS salts for salting-out are NaCl and MgSO_4_ in a 4:1 ratio, and this ratio is often commercially available in “salting-out” kits [53,57,75,82,87]. NaCl alone (600 mg) can increase the signal-to-noise ratios up to 2.5 times higher than the combined use with MgSO_4_ [81]. The use of buffers can increase the recovery of acid-sensitive PFAS precursors. A “safe” starting point could be 5 g of sample +0.5 g of disodium citrate sesquihydrate +1 g of sodium citrate +4 g of MgSO_4_ and 1 g of NaCl [53,74].

Despite different validated methods for the extraction of PFAS with QuEChERS techniques, there are some limitations that must be noted. Sorbent selection represents a critical choice in all QuEChERS-based extraction techniques, especially in PFAS, considering structural classes as different sorbent materials exhibit compound-specific adsorption characteristics which can impact accuracy [53]. PSA serves as a weak anion exchanger designed to remove organic acids, fatty acids, and polar interferences (e.g., sugar) through electrostatic interactions [81,122]. Therefore, PSA’s ion-exchange properties can retain ionizable PFAS (e.g., PFCAs, PFSAs), and they decrease recoveries if used in high quantities (e.g., 400 mg) [71,76,78]. It can cause significant loss of acidic PFAS (with recoveries falling below 50% for some carboxylates), requiring a reduction in its amount or its replacement with neutral polymers like PS-DVB [53,78].

Anionic PFAS, such as PFPAs and 8:2 PAP, are often completely lost during SPE or dSPE clean-up involving a weak anion exchanger (WAX) or GCB [123]. Recently, the use of other sorbents (PS-DVB, MWCNTs), magnetic nanomaterials like Fe_3_O_4_-TiO_2_, Florisil, and carbon X can target specific matrix interferences that traditional sorbents like PSA or C18 may fail to remove without analytical losses [53,71,75,77,78]. Multiple sorbents can produce interactive effects distinct from individual components and individual interactions with solvent and matrices [73]. The use of buffering agents (e.g., disodium citrate sesquihydrate, sodium acetate) can help to increase recovery of acid-sensitive PFAS precursors (N-MeFOSAA and N-EtFOSAA, FTS) [47,86].

In the next sections, we will discuss general strengths and challenges for each matrix with the aim of guiding ready-to-validate method development for a specific food.

#### 3.3.3. Aquatic Animals’ Products (Fish, Crustaceans)

As mentioned in Section 3.3.1, aquatic animals’ products are the most studied matrices for the extraction of PFAS with the QuEChERS protocol. There are already validated methods that analyze the EU-regulated PFAS, in accordance with EU regulation performance criteria, in fish muscle as well as for NTS analyses [15,51,74,81]. For fresh tissue, the sample/solvent ratio ranges from 1:2 to 1:11 (*w*/*v*) [15,48,51,53,57,71,73,74,75,78,83,86,92], while up to 1:66 was used for freeze-dried samples and NTS analyses [73,81]. Mechanical homogenization, mechanical mincing and cryogenic grinding are used to reduce the interaction of PFAS–matrix and to increase the homogenization of the sample [53,57,71,74,86,123]. Sonication is also used to increase recovery and precision, both pre and post-extraction, and up to one hour of sonication at 30 °C was used to have better analytical parameters [73,83]. For fish and crustaceans, most authors applied the standard acidified ACN/EN salt template described in Section 3.3.2. Proteins and lipids are the primary contributors to the matrix effect and PSA (0.012–300 mg), GCB (0.012–300 mg), C18 (0.012–200 mg), followed by Envi-CARB (5–500 mg), UCT (150 mg), Florisil (100), PS-DVB (50 mg), Fe_3_O_4_-TiO_2_ (20 mg) and CarbonX (0.001 mg) can help to reduce these effects [53,74].

Liu et al. (2024) reported that more than 20 mg of C18 can decrease the recoveries of FTS, while less than 10 cannot be sufficient to remove lipid [53]; Gao et al. (2018) used 100 mg of C18 (+200 mg of PSA +100 mg of Florisil +50 mg of Envi-carb) to analyze legacy and emerging analytes (FTS included) [75]. An alternative strategy within QuEChERS workflows is to add an SPE clean-up step, which can efficiently remove lipids while preserving PFAS recoveries [76]. Filtration passage is also common (Table S5) with nylon filters at 0.22 μm, which is preferred because they can be used with no teflon rubber packaging that is a possible source of contamination [71,75,81]. Other materials involved in filtration are PES, PVDF, PP, and paper filters [15,48,78,83,86]. Crustaceans were validated in three sources and represent an analytical challenge despite the highest level of PFSA reported with co-occurrence and a PFOA content higher than MLs limits [15,71,81]. In particular, wild shrimp contained more PFAS than farmed [15]. Regarding matrix effects, FOSA and FOSE compounds can coelute with hydrophobic interferences and cause signal suppression, while FTS and FOSAA can have matrix enhancement [53,73,74]. The length of the PFSA’s chain seems to influence suppression (≤10 carbons) or enhancement (>10 carbons) of the signal [75].

Few methods are currently available for PFAS determination in crustaceans [15,71,81], and most sources focus on fish filets (muscle) rather than other tissues (viscera, fish organs) that might contain higher concentrations of PFAS. There is a clear need for additional methods targeting these matrices.

#### 3.3.4. Terrestrial Animal Tissues (Muscles, Offal)

All reviewed methods that include terrestrial animal tissues (poultry, swine, bovine, goat muscle) also analyze fish muscle and/or crustaceans (Table S4). Most of the considerations described for aquatic animal products can also be extended to terrestrial animal muscle, considering the higher MLs permitted for these matrices. The same does not apply to offal, which has been validated in four studies and is considered a challenging matrix [15,48,71,83]. Three of them used HRMS that performs better than QqQ because cholic bile acids are isobaric interferences for PFOS and can cause false positives in low resolution [57,82,123]. Gallocchio et al. (2022) separate PFOS from taurodeoxycholic acid chromatographically [48].

While a simple QuEChERS extraction may be sufficient for muscle tissue, offal requires a more aggressive purification. Methods that included offal typically used higher PSA and C18 quantities (≥150–300 mg PSA and 150–500 mg C18) that are used specifically to remove fatty acids and cholic acid included in these matrices [15,81]. dSPE is often conducted twice or followed by a SPE scheme, such as 500 mg ENVI-Carb (or Bond Elut Carbon S) followed by 500 mg PFAS-WAX cartridges [15,81]. Regarding matrix effect, pork muscle contains higher lipid levels that can interfere [123]. Offal has signal suppression for next-generation PFAS like HPFO-DA [123]. All methods that include offal have a filtration step prior to analysis, and therefore, filtration is highly suggested (Table S4).

#### 3.3.5. Eggs

Eggs represent not only one of the major dietary contributors to human PFSA exposure, but also a challenging matrix. All methods in this review that include offal in their matrix panel also analyze eggs, reflecting the related analytical issues [18,48,71,83]. Two other authors analyze eggs but with no offal [57,81]. However, eggs have lower MLs than offal, which increases the analytical challenge. Cholic acid and bile acids are also present in eggs and can cause false positives in QqQ for the PFOS analyte [57]. For purification purposes, a two-step SPE (500 mg of ENVI-Carb and WAX cartridge) can be useful to remove fatty acids and pigments from this matrix [15].

Typical workflows use a fully buffered EN-type salt packet (≈0.5–1.5 g disodium citrate sesquihydrate, 1 g sodium citrate, 4–6 g MgSO_4_, 1 g NaCl). The dSPE phase includes PSA (to remove organic acids, 150–300 mg), C18 (for lipids, >150 mg), and GCB (for pigments) [15,57,81].

Alternative clean-up strategies for egg extracts included freeze-out steps and membrane filtration or ultrafiltration to reduce the lipid load before injection [57,81,83,123]. However, these passages can retain long-chain PFAS or certain PFAS (HFPO-DA, NEtFOSAA, NMeFOSAA) that show poor sensitivity or low recoveries in eggs compared to beef or fish [15,57]. Eggs can induce both ion suppression for long-chain PFAS and ion enhancement for others [15,85].

#### 3.3.6. Dairy and Infant Foods (Milk, Infant Formula)

Only Abafe et al. (2021) validated a QuEChERS method for infant foods successfully, while different authors developed a QuEChERS method for milk [18,48,71,76,84,85,87]. Considering the high-protein content, especially albumin in milk, ACN or MeOH are preferred [15,48]. Regarding salts used for the extraction salting-out step, NaCl (600 mg) can increase the signal-to-noise ratio up to 2.5 times higher than with other salts [81]. dSPE clean-up is performed combining PSA (150–300 mg) and C18 (150 mg), sometimes in addition with GCB or magnetic sorbent (50 mg magnetite–silica and 40 mg ZrO_2_), to reduce residual lipids [76]. Further passages may not be needed. Gallocchio et al. (2022), Abafe et al. (2021) and Shi et al. (2023) validated a confirmatory method for milk with QuEChERS protocol without any SPE step on QqQ in accordance with Commission Decision 2002/657/EC [48,84,87]. Other authors used a multistep clean-up scheme for the simultaneous analysis of milk with other matrices [18,71,85]. Regarding matrix effect, an ion suppression/enhancement is reported for different PFAS (e.g., PFBS +29%, PFDoA −45%) that can increase the difficulties to obtain acceptable performance criteria [48,87]. Milk contains later-eluting compounds that can interfere with long-chain PFAS [85].

#### 3.3.7. Plant Products (Fruit, Vegetables, Rice, Coffee, and Vegetable Oil)

As mentioned in Section 3.3.1, plant products are underreported and represent one of the most difficult matrices for the PFAS extraction with QuEChERS protocols. They are characterized by different chemical profiles, ranging from the high-water content of berries to the high-fat level found in olives. However, QuEChERS protocols have been applied successfully to different fruits and vegetables [15,47,71,72,73,74,78,80,81,84,86].

Solvent ratio ranged from 1:1 to 1:6 for fresh samples and 1:15–1:110 for the freeze-dried [15,70,71,72,73,77,80,82]. Sonication procedure, from brief pulses to one hour, can also increase the recoveries and separate the matrix from the pigments, especially in olive fruits [48].

Plant products shared the same basic QuEChERS design but required greater adaptation of sorbent blends due to heterogeneity. Buffered ACN (often with 1% FA or AA) and EN-type salts combined with PSA (90–300 mg) and GCB (60–150 mg), or polymeric carbon sorbents (ENVI-Carb), can remove chlorophyll and phenolic compounds present in leafy vegetables and fruits while preserving PFAS recoveries [70,72,77]. This is extremely important in cranberries, where anthocyanins can cause matrix suppression [70,72]. Triglycerides and lipid content of olive fruits and edible oils require up to 400 mg GCB and 120–200 mg PSA or C18 for reducing the matrix effect below 10% or a combination of ENVI-Carb/C18/GCB or Agilent EMR-Lipid dSPE before instrumental analyses [70,82]. For rice, the extraction solvent was a mixture of water: ACN (1:2, pH ≈ 2.4) with NaOAc, MgSO_4_, and NaCl and 300 mg PSA + 150 mg GCB [79].

For coffee and other brewed beverages, solvent systems based on ACN (often with 0.1–1.5% FA or AA) and the 4 g MgSO_4_/1 g NaCl salt combination are used, with dSPE recipes such as MgSO_4_ + PSA or more complex ENVI-Carb/C18/GCB mixtures to address caffeine, Maillard products, and other components [79].

#### 3.3.8. Take Home Messages

There is no “best solution” for all the matrices, and all the methods must be optimized for instruments, PFAS, and matrix panels, the aim of the method. A common start for the extraction procedure could be acidified ACN + EN-type salt template. Use ACN with 0.1–1% FA/AA/HCl and a standard EN-type salt packet per 5 g equivalent (≈0.5–1.5 g disodium citrate, 1 g sodium citrate, 4–6 g MgSO_4_, 1 g NaCl). Homogenization, sonication, and the phase separation step (centrifugation) are passages that can help before the dSPE step. Poor tissue disruption and incomplete phase separation are major sources of imprecision and matrix effects. Olive oil, eggs, and offal can be a challenging matrix. For dSPE steps, a common start could be PSA/C18, which can be sufficient and increase or diversify sorbents (GCB, ENVI-Carb, PS-DVB, EMR-Lipid, WAX) only when recoveries, LOQs, or matrix effects fail regulatory or validation criteria. Reserve heavy sorbent loads and two-step SPE (e.g., ENVI-Carb + WAX) for the most challenging matrices (offal, eggs, olives) and consider HRMS for these matrices. Prefer nylon or PES if filtration is needed.

### 3.4. Instrumental Analyses and Quality Assurance/Quality Control (QC)

#### 3.4.1. Instrument Choice: LC-MS/MS or HRMS

Across the reviewed methods, ESI in negative mode was used almost exclusively, reflecting the acidic nature of PFAS and their efficient deprotonation under typical LC conditions. LC-MS/MS is the most common instrument for the quantitative PFAS determination in food and provides broader linear ranges for certain PFAS (e.g., FOSAAs, HFPO-DA) [57]. These performances require the use of isotopically labeled compounds [57,81].

HRMS offers clear advantages for suspect and NTS, retrospective analyses and can cope with isobaric compounds more easily, which can lead to false positives (cholic acid for PFOS) [51,57]. Taylor and Sapozhnikova developed a QuEChERSER method (where the last two letters stand for efficient and robust), and they compared HRMS and QqQ directly for the analyses of the same samples. While HRMS can differentiate isobaric compounds, QqQ requires changing the PFOS transition from *m*/*z* 499 > 80 to 499 > 99 in egg extracts to avoid false positives [57].

HRMS is the only effective tool for identifying unknown or previously unlisted PFAS. The use of HRMS allowed researchers to detect PFPrA in an egg sample for the first time, a compound that would have remained invisible to a targeted LC-MS/MS method [81]. Furthermore, HRMS is essential for branched isomers that were resolved in five studies, with four of them in HRMS [15,57,71,73,74]. Short-chain PFAS can hardly be detected in QqQ because some of them (PFBA, PFPeA) have one ion transition and can be difficult to confirm without a secondary ion [71,81].

Both instruments play complementary roles. LC-MS/MS is useful for analyzing already regulated PFAS and increasing the matrices validated for regulatory purposes, whereas HRMS is used to investigate and expand the analytical scope for emerging PFAS or unexplained signals in particularly challenging matrices.

#### 3.4.2. Columns and Mobile Phases

**Figure 3:**
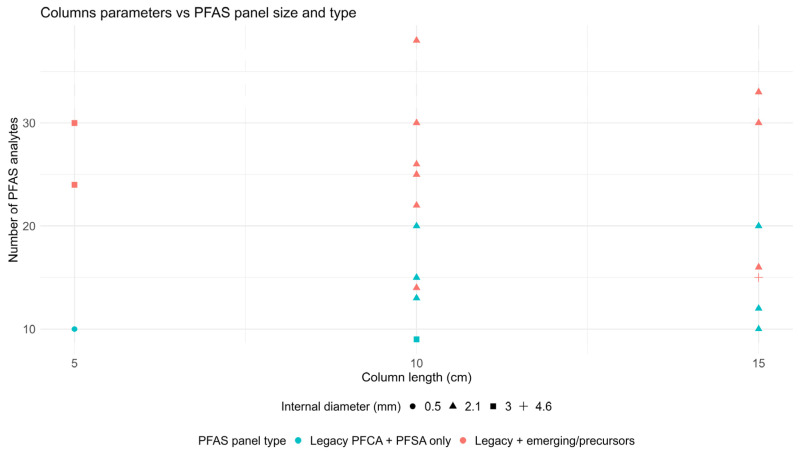
Column parameters vs. PFAS panel size and type. Scatter plot showing the number of targeted PFAS analytes as a function of LC column length, with symbols indicating column internal diameter and colors distinguishing PFAS panel classes. Methods were classified as “legacy PFCA + PFSA only” when targeting PFCA and PFSA exclusively, and as “legacy + emerging/precursors” when the panel also included PFECA, FTS, FOSA and related compounds, and/or other emerging PFAS. The 2.1 × 100 mm columns represent the most common column used for the analysis of PFAS.

For most of the reviewed methods, PFAS were separated almost exclusively on reversed-phase C18 columns with lengths between 50 and 150 mm, internal diameters of 2.1–3.0 mm, and particle sizes from sub-2 µm (1.7–1.9 µm) to conventional 2.5–5 µm (Figure 3, Table S3). Columns of 50–150 mm × 2.1 mm are the dominant configuration across food matrices, and this makes methods more transferable across laboratories and agencies. These columns provide sufficient resolving power to baseline-separate homologues and branched/linear isomers within 10–20 min runs [15,47,51,53,57,70,71,72,76,77,78,79,81,84,85,86,87,112]. The length of the column (>50 mm) is important only when the PFAS panel is n > 30. If the number of PFAS to be analyzed is fewer, improvements in sample preparation or instrumental methods can be sufficient even for the analyses of PFECAs. Standard C18 phases can struggle with ultra-short-chain PFAS, and this can be one of the reasons why these compounds are not studied enough. Shorter columns are used where faster analyses are prioritized over PFAS panels [73,80,82,83,124]. Sub-2 µm particles deliver narrow peaks and high plate counts at UHPLC pressures, whereas 2.5–5 µm particles of similar dimensions offer greater robustness and compatibility with conventional HPLC systems for routine food-monitoring capacity [15,47,51,53,57,71,72,75,76,77,78,79,81,84,85,87,112,125].

Because PFAS leach from LC hardware, most methods include a PFAS delay column and/or a short guard column (typically 2.1 × 5–50 mm) between the mixer and analytical column to trap system-derived PFAS and protect the main column from matrix lipids and proteins over long injection sequences [15,47,71,74,76,78,79,80,85]. These columns help to mitigate the contamination of PFAS into LC systems or delay the elution of high lipid and protein content of complex food extracts. Genualdi et al. noted that using a guard column with a smaller internal diameter (e.g., 2.1 × 5 mm, 1.7 μm) was more effective at preventing matrix breakthrough to the analytical column than larger versions [71].

The selection and composition of the mobile phase can influence ionization efficiency, peak shape, and the resolution of analytes from complex matrix interferences [48,77]. Mobile phases were made by an aqueous phase (A) of water with 2–20 mM ammonium acetate or ammonium formate, sometimes with 1-methylpiperidine or a small amount of formic/acetic acid, and an organic phase (B) of methanol or acetonitrile containing the same volatile buffer and/or 0.1–0.3% acid [48,51,70,71,74,75,82,84].

Methanol is generally preferred when broad separation of PFAS homologous series and isomers is needed, while ACN is often selected for vegetable screening, high PFAS panels such as NTS applications, where lower backpressure is advantageous [48,74,76,77,81,85]. Acid conditions (0.1% *v*/*v*) of the mobile phases can increase the peak symmetry of short-chain carboxylic acids like PFBA, the performance of early-eluting compounds, and sensitivity in ESI. Ammonium acetate is the most common additive; pH adjusting (2.4 to 3.5) using formic acid can stabilize short-chain PFCAs [47,70,73,123].

Gradient programs started with a high aqueous percentage (typically 90% to 95% Phase A) to allow the initial retention of polar PFAS, followed by a linear increase in the organic phase to elute long-chain compounds [51,74,76]. Flow rates for methods with columns of 2.1 mm internal diameters are typically maintained between 0.2 and 0.4 mL/min, while some specialized micro-HPLC methods utilize much lower flow rates of approximately 20 µL/min [15,82,83].

Taken together, the data in Figure 3 suggest that laboratories analyzing broader PFAS panels (>30) tend to adopt longer columns (>100 mm) with an internal diameter of 2.1 mm. Shorter columns (50 mm) and larger particle sizes (2.5–5 µm) may compromise the resolution of complex PFAS panels, and methods developed with these columns are less frequent. Standard C18 columns can struggle to retain ultra-short-chain PFAS like PFBA and PFPeA that can elute near the solvent front, while columns with polar embedded groups enhance the retention of these early-eluting acids. Few studies systematically assess how changes in organic modifier, buffer strength, or pH translate into gains in detection limits, selectivity for isomeric species, or tolerance to matrix effects.

#### 3.4.3. Quality Assurance and Quality Control

PFAS analyses can be tedious, considering the necessity to monitor analytical performances, PFAS leaks from the instrument, strong matrix effects, and the possible presence of PFAS in blanks. Most of the reviewed PFAS methods implemented QA/QC practices (Table 1 and Table S6). Isotopically labeled internal standards or surrogates were used in all the sources reviewed to correct one or more parameters such as extraction losses, volumetric deviations, and matrix effects (suppress or enhance) [18,47,48,51,53,57,70,71,72,73,74,75,76,77,78,79,80,81,82,83,84,85,86,87]. Both extracted internal standards (EIS) and non-extracted internal standards (NIS) were used to monitor the entire extraction efficiency and evaluate ionization variations. Contamination control was also widely addressed, with 83% of methods reporting at least one blank strategy (procedural and/or solvent blanks per batch), frequently combined with PFAS-free hardware, delay or guard columns, and replacing PTFE or Teflon with PEEK or HDPE to mitigate system background [15,16,50,71,72,74,75,78,79,81,122,123]. When these strategies were not possible, the signal of the blanks was subtracted from the signal of the samples to calculate the concentrations [78]. About half of the methods (46%) incorporated matrix spikes and/or duplicate samples per batch, and 20% performed continuing calibration checks (ICV/CCV) at the beginning/end of the batch or every 20 injections, supporting ongoing verification of calibration stability [47,48,53,57,73,74,76,77,80,86,87]. Matrix effects were explicitly evaluated in 79% of the studies, mostly by comparing slopes of matrix-matched and solvent calibration curves or by calculating matrix factors/SSE% or by direct comparison of peak areas [48,51,53,57,70,71,73,74,76,77,78,79,80,82,83,84,85,87]. LOQs were calculated by an S/N ratio of 10, as 3.3 × LOD or as the lowest spiking level meeting predefined performance criteria [15,47,48,72,74,75,76,79,80,81,83,84,86]. Regarding accuracy and precision, the biggest problem of QuEChERS extraction is maintaining them in an acceptable range for all the analytes. Several authors dropped some PFAS (e.g., PFNS, PFDS, FBSA, NFDHA, PFOS) in the PFAS panel analyzed because it was impossible to maintain recovery or precision in the range of predefined performance criteria [15,57,73,85]. Regarding precision, it was reported as RSD, and only some sources explicitly reported precision calculated over different days (intermediate precision, RSD_R_) [53,70,84,85,87,113]. Only 20% of the methods used SRMs to verify accuracy or assess matrix effects, 8.3% reported formal robustness studies (e.g., varying sample weight or chromatographic conditions), 12.5% provided expanded uncertainties (often using U = k × u or Nordtest-type approaches), and 16% reported participation in interlaboratory studies [15,48,57,71,73,74,75,87]. Finally, only 16% of the methods explicitly addressed the chromatographic separation of linear and branched PFAS isomers, mostly with HRMS [15,57,71,74].

#### 3.4.4. Compliance with European Regulatory Frameworks

QuEChERS protocols can obtain performance criteria that are required by guidelines or regulations all over the world. The primary performance criteria that must be addressed in PFAS analytical method validation include linearity, accuracy (trueness), precision (repeatability and reproducibility), sensitivity (LOD/LOQ), selectivity (specificity), and the characterization of matrix effects. Acceptance criteria for these methods are often dictated by regulatory guidelines such as Commission Decision 2002/657/EC, the AOAC SMPR, or EPA Method 1633 [15,30,51,74,80]. The transition from the more permissive Commission Recommendation 2010/161/EU (which allowed 70–120% recovery) to the current EU 2022/1428 standard (requiring 80–120% trueness and ≤20% RSD) represents a significant increase in validation difficulty [68,83]. The results of the classification procedure between “Explicitly claimed compliant”, “Non-compliant”, and “Apparently compliant” are presented in Table S6, and the analytical performance of the method can be seen in Tables S6 and S7. Recent methods explicitly align their validation with Regulation (EU) 2023/915 MLs and Regulation (EU) 2022/1428, reporting LOQs at or below the MLs, trueness within 80–120%, and RSDs ≤ 20% [15,51,74]. However, only two reported branched/linear differences and one reported both branched/linear quantification and expanded uncertainties as required by Regulation (EU) 2022/1428 [15,51,74]. A larger group of studies can be considered “apparently compliant” in their analytical parameters for the four regulated PFAS and meet the numerical criteria in Table 5 of Regulation (EU) 2022/1428 and/or (EU) (2022/1431) for the corresponding matrices [48,57,73,75,76,78,83,84,87]. Some of them reported expanded uncertainties [48,87] while others differentiate branched/linear PFAS [57,73].

Finally, there are different “non-compliant” methods that are valid but were developed before the actual European regulations, outside the EU, and for research purposes [71,80]. Even if the EU Recommendation (2022/1431) is not mandatory, the LOQ limits can be really hard to obtain, especially for fruits and vegetables, and represent one of the most common causes of non-compliance [47,70,71,72,77,79,80,82,85,86]. Using EU regulation as a reference has important limitations outside Europe. Laboratories in other regions could validate methods according to alternative guidelines (e.g., EPA, GB 5009.253–2016) and could terminate the validation process once they have met their own predefined objectives, which are not necessarily aligned with EU requirements [126,127]. Furthermore, the geographical bias limits the global applicability of the findings.

However, EU regulation on PFAS in food is among the most stringent worldwide, and for this reason, we adopt it as the primary benchmark for evaluating analytical methods for performance criteria.

Older studies utilized remain robust, but a re-validation is needed for the tighter acceptance criteria. The branched/linear PFAS question remains a problem that needs more solutions.

#### 3.4.5. Take Home Messages

For the four regulated PFAS, LC-MS/MS methods achieve the performance criteria of Regulation (EU) 2023/915 and 2022/1428 when appropriate QA/QC is applied, and isotopic standards are available. HRMS adds unique value for branched/linear isomers. Across the 24 sources reviewed, analytical parameters compliant with EU regulation can be obtained for the regulated PFAS (PFOS, PFOA, PFNA, and PFHxS) in high-protein matrices such as fish, meat, eggs, and milk. The predominant cause of non-compliance is insufficient LOQ, particularly for fruits and vegetables, where the 0.001 µg/kg targets in Recommendation 2022/1431 remain technically challenging. This gap reflects the combined effect of strong matrix effects in plant matrices, background contamination, and instrumental limits, rather than a fundamental inability of QuEChERS-based methods to deliver accurate and precise data. Quantification of branched/linear isomers remains an open challenge for regulatory purposes.

### 3.5. PFAS Analyzed

**Figure 4:**
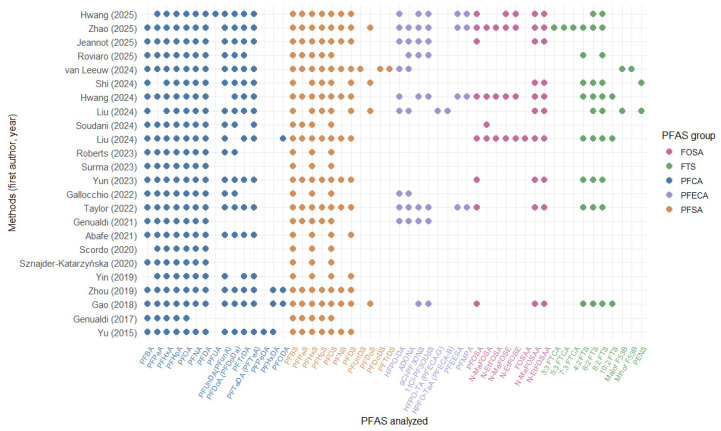
Span and composition of PFAS panels in the 24 validated LC–MS/MS methods reviewed. Each row represents a method (ordered chronologically), and each point marks an individual PFAS included in the corresponding analytical panel, with point position along the *x*-axis indicating the specific compound. Colors identify chemical class (PFCA, PFSA, PFECA, FOSA derivatives, FTS, or other PFAS). From 2015 to 2025, methods have been developed to increase the range of PFAS analyzed, considering the new compounds discovered. Early protocols (2015–2018) targeted relatively small panels dominated by PFSAs and PFCAs, whereas recent methods (2022–2025) consistently include FTSs, PFECAs and FOSA, often exceeding 30 analytes. Despite the temporal expansion of the PFAS panel, there are persistent gaps, such as the limited inclusion of PFECAs and short-chain PFAS [15,47,48,51,53,57,70,71,72,73,74,75,76,77,78,79,80,81,82,83,84,85,86,87].

The analysis of PFAS in food matrices with QuEChERS protocols has expanded from nine compounds to more than 40 simultaneous quantification (Figure 4, Table S6) [70,74]. Almost all studies prioritize the four compounds frequently regulated that are 4-EFSA-PFAS: PFOS, PFOA, PFNA, and PFHxS [15,51,74,81,83,86]. These four compounds appear in the major part of the reviewed studies. The inclusion of branched and linear isomers for compounds like PFOS is also highlighted as essential for accurate quantification in complex animal tissues [15,74]. PFCAs represent the most frequently analyzed PFAS class across all food matrices, present in almost all reviewed studies. This homologous series features a perfluorinated carbon chain terminated by a carboxylic acid functional group (-CF_2_COOH), with chain lengths ranging from C4 to C18 carbons. Short PFCAs (C4–C7) and long-chain compounds (PFHxDA, PFODA/PFOcDA) were successfully detected and quantified in different food matrices [51,70,76,77]. Researchers developed QuEChERS protocols that targeted precursor compounds that can transform into persistent PFAS, such as FTSs ranging from 4:2 to 10:2, and perfluorooctane sulfonamido acetic acids like (N-MeFOSAA and N-EtFOSAA) [73,81]. Investigations were conducted into matrices like rice and brewed capsule coffee that have also included sulfonamides and sulfonamidoethanols such as FOSA, N-MeFOSE, and N-EtFOSE [47,79]. Also, “next-generation” industrial alternatives designed to replace legacy chemicals, such as ether acids like GenX (HFPO-DA), ADONA, and NaDONA, have been analyzed successfully [15,51]. Polyfluoroalkyl ether sulfonates, such as 6:2 and 8:2 Cl-PFAES (the components of F-53B), have emerged as significant contaminants in aquatic and marine environments. [53] While some methodologies remain focused on a core set of 9 to 15 legacy PFAAs to meet specific regulatory benchmarks in fish or milk, others adopt high-capacity target lists, such as the 40 analytes specified in EPA Method 1633, to provide a broader view of contamination [48,74,83,86]. There are different strategies to expand PFAS panels and to detect compounds that were not included in the original target list. Jeannot et al. (2025) combine HRMS with Kendrick Mass Defect (KMD) plots to identify previously unlisted PFAS, in fact PFAS of the same homologous series align horizontally in KMD space [81]. Another approach is the use of “sandwiched injection”, which can mitigate solvent-peak distortion for early-eluting, short-chain PFAS and improve their detection and quantification [74]. Another strategy that recent methods use to improve the analysis of short-chain PFAS is EMR, which can remove lipid and improve the short-chain PFAS signal [53,74].

## 4. Conclusions and Perspectives

This review has limitations. Analytical methods published after December 2025 were not captured by the search strategy and are therefore not included; only methods explicitly describing a QuEChERS or d-SPE step with salts were considered. Methods that analyze more types of analytes or non-food matrices were not included. However, there are methods that do not meet the eligibility criteria of the selection process but allow the analysis of different PFAS in multiple matrices without the use of salting-out mixtures [123]. Then, considering heterogeneity in food matrices and PFAS panels, as well as report validation parameters, a formal meta-analysis is precluded. Other than a customized RoB, no formal tool (e.g., risk-of-bias or certainty-of-evidence framework) was applied to grade the methodological quality of individual studies, and the assessment of robustness and generalizability of the reviewed methods is therefore qualitative.

This review shows that the QuEChERS extraction can be used for PFAS determination in LC-MS/MS and HRMS (Table 2) from more than 45 PFAS panels (Figure 4). Methods are converging in matrices analyzed (Figure 2), salts, chromatographic conditions (Figure 3), and QA/QC practices (Table 1). The sorbent choice is not included in this “comfort zone” and remains matrix/PFAS/method dependent without a common strategy. At the same time, these methods have struggled to analyze certain PFAS (Figure 4) or some matrices (Figure 2) for the past 10 years. Critical aspects remain commercially available isotope standards and SRMs that can limit study comparability as assessed by RoB. Recent publications demonstrate that vegetables, berries, and packaged foods can be relevant contributors to dietary exposure, especially for C4–C7 PFCAs and packaging-derived precursors. Extended PFAS panels and advanced HRMS instrumentation are needed to capture these fractions. The major part of the current literature cannot demonstrate compliance with new EU regulations and therefore cannot be directly used for food monitoring, and the quantification of branched/linear isomers remains an open challenge, as well as short-chain PFAS.

**Figure 5:**
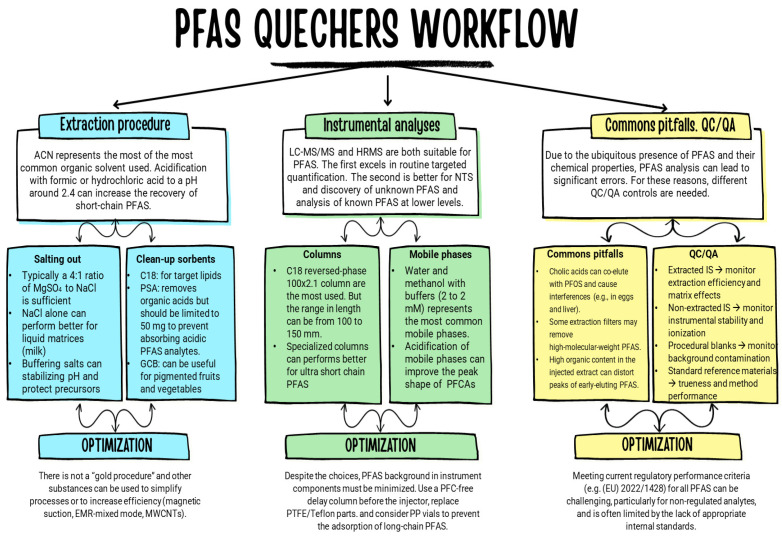
Overview of the PFAS QuEChERS workflow with key steps in extraction, instrumental analysis, and QC/QA, with common pitfalls and optimization strategies for PFAS determination in food matrices.

Future work should therefore move beyond incremental adaptations of existing protocols and focus on targeted optimization and harmonization efforts. A good strategy could be to have a common procedure of validation for RSD (RSD and RSDR), LOQ, robustness, uncertainty, and proficiency test participation. Common performance criteria will not only improve data quality for current regulatory targets but also serve as an incentive for harmonizing future legislation. NTS by HRMS is a crucial complement for PFAS in food. Target analyses can be used in food safety labs, but they are blind to the vast majority of unknown or transformation products that may be present in food. NTS workflows offer the unique capability to uncover previously unmonitored PFAS, to track changes in industrial usage over time, and to flag candidates for future regulatory consideration. Future method development should prioritize integration between targeted quantification and NTS (e.g., shared sample preparation and harmonized QA/QC and reporting criteria) so that regulatory compliance data can be systematically complemented by broader PFAS fingerprinting and suspect/non-target discovery in food. Future work should prioritize plant-based matrices and short-chain PFAS, also. However, these two parameters combine difficult matrices with difficult analytes that are challenging to detect analytically. For method developers, the following practical recommendations emerge from this review (Figure 5): (1) acidified ACN-based QuEChERS with a 1:1–1:3 (*w*/*v*) sample-to-solvent ratio represents a starting point for most food matrices; (2) dSPE sorbent selection should be guided by matrix lipid and pigment content, C18/PSA combinations for animal tissues, and GCB-free protocols for plant matrices to avoid PFAS adsorption losses; (3) isotope-labeled internal standards covering all PFAS subclasses are essential for accurate quantification; (4) HRMS should be considered when the analytical scope includes PFAS precursors or difficult matrices that contain isobaric compounds or ultra-short-chain species; and (5) future method development should prioritize plant-based matrices, short-chain PFAS (C4–C6), branched/linear isomer separation, and geographically underrepresented regions, particularly Africa and South America.

## Figures and Tables

**Table 1:** Quality assurance and quality control (QA/QC) strategies reported across the sources.

Strategies for Monitoring PFAS Analyses	%	Notes
Isotope utilized	100	To validate the method, the authors used isotope standards when possible. If not commercially available, authors used a surrogate instead.
Blank strategy during analyses	83	Common strategies include procedural or/and/or solvent blanks for each batch or when needed. If the instrumental system leaches PFAS, the signal of blanks was subtracted from the calibration curve. The guard and delay columns are also applied.
Spikes/Duplicates during analyses	46	One/two matrix spiked standards in each batch or after some injections.
Continuing Calibration Verification (ICV/CCV)	20	At the beginning and at the end of the batch, or every 20 injections.
Matrix effect assessment	79	Almost calculated by the ratio of the slope of the matrix-matched calibration curve and the slope of the calibration curve of the standard solution in solvent at the same spiking range. Some authors compare the area.
Recovery/trueness (%)	95	Most sources calculate recovery as the ratio between the measured concentration in spiked samples/spiked concentrations.
Precision (%RSD)	83	Precision was calculated as the population standard deviation divided by the population mean. It is calculated by replicates (from 3 to 7), often in a single day. Some sources report inter-day precision evaluated from 3 to 5 days.
LOD/LOQ	95	Calculated from the S/N ratio of 10, 3.3 × LOD (3.3 times the standard deviation of the blank), or some studies report the LOQ as the lowest spiked level that respects performance criteria.
SRM	20	Reference material was used for evaluating the performance of the method or to calculate the matrix effect.
Robustness	8.3	Reported by 2 sources. Calculated by varying sample weight, particle size of chromatographic columns, or mobile phases.
Expanded uncertainties	12.5	The expanded measurement uncertainty was calculated with the U = k × u, where u is the combined standard measurement uncertainty and k (2) is the covering factor, or through the Nordtest Approach.
Participation in interlaboratory studies	16	Four sources participate in interlaboratory studies.
Branched/linear separation	16	Separation between branched and linear isomers of PFAS was performed by four sources. Three of them were obtained by HRMS.

**Table 2:** Summary of QuEChERS-based LC–MS/MS and HRMS methods for PFAS determination in food matrices analyzed in this study. The table reports, for each study (S), the food matrix, country, number of PFAS analyzed analytes (N), extraction solvent, clean-up sorbent(s), instrument (LC–MS/MS or HRMS), limits of quantitation (LOQ range), recovery (R%) and precision (RSD) ranges, number of isotope-labeled internal standards (N_IS_), application of matrix-matched calibration, and classification of European compliance with current performance criteria.

S	Matrix Type	Country	N	Extraction Solvent	Salting-Out Salt and Clean-up Sorbent	Instrumentation	LOQ	R%	RSD	N_IS_	Matrix Matched Calibration	Regulatory Compliance
[51]	Fish muscle, Cow muscle	Italy	20	ACN with 1.5% AA and water	sodium chloride; magnesium sulfate; magnesium sulfa e; PSA; C18	Orbitrap Q-Exactive (Thermo Fisher Scientific, Bremen, Germany)	0.1–1.0 μg/kg	80–120%	RSD ≤ 20%	20	Not performed	Explicitly claimed compliant
[73]	Fish muscle, Vegetables	USA	24	ACN:H_2_O	sodium chloride; magnesium sulfate; PSA; C18; ENVI-Carb	X500R QTOF (SCIEX, Framingham, MA, USA)	0.67–10.0 ng/g	73 ± 131% (TU), 51 ± 125% (ELAP) (N-MeFOSAA, N-EtFOSAA were excluded)	RSD% 5–15%; some precursors (FTSs, N-MeFOSAA, N-EtFOSAA, PFOSA) extraction standard recoveries were <30% or >150%, so no RSD could be established	19	Not performed	Apparently compliant
[81]	Fish muscle, Poultry muscle, Swine muscle, Cow muscle, Goat muscle, Crustacean, Eggs	France	25	ACN:H_2_O + 1%FA	sodium chloride; magnesium sulfate; C18; GCB	QqQ 6495c (Agilent Technologies, Santa Clara, California, USA), Orbitrap Q-Exactive (Thermo Fisher Scientific, Bremen, Germany)	Not reported	Not reported	Not reported	16	Not performed	Non compliant
[57]	Fish muscle, Poultry muscle, Swine muscle, Cow muscle, Eggs	USA	33	5 mL of 4:1 (*v*/*v*) acetonitrile/water	sodium chloride; magnesium sulfate; PSA; C18; CarbonX	SCIEX 6500 QTRAP™ MS/MS system (SCIEX, Framingham, MA, USA); Q-Exactive Plus Hybrid Quadrupole-Orbitrap™ system (Thermo Fisher Scientific, Bremen, Germany)	HRMS: 0.9–938.3 ng/g QqQ: 0.9–916.9 ng/g	70–120%	RSDs ≤ 20%	20	Performed	Apparently compliant
[80]	Vegetables, Fruits	Australia	30	ACN:H_2_O	sodium chloride; magnesium sulfate	Agilent 6470 triple quadrupole mass spectrometer (Agilent Technologies, Santa Clara, CA, USA)	0.0245–3.6323 ng/g	71.0–128.1%	RSDs ≤ 20%	22	Not performed	Non compliant
[87]	Infant formula, Milk	South Africa	15	ACN:H_2_O	sodium chloride; magnesium sulfate	PerkinElmer^®^ QSight™ 220 (PerkinElmer, Waltham, MA, USA)	5–50 ng/kg	60–121%	5–28%	2	Performed	Apparently compliant
[83]	Fish muscle, Poultry muscle, Swine muscle, Cow muscle, Goat muscle, Eggs, Liver, Vegetables	Poland	10	ACN 1.36% AA and water	sodium chloride; magnesium sulfate; ENVI-Carb	QTRAP 5500 (SCIEX, Framingham, MA, USA)	0.013–0.028 ng/g	82–102%	RSDs ≤ 20%	2	Performed	Apparently compliant
[77]	Vegetables, Fruits	China	20	ACN with 1% FA	sodium acetate; magnesium sulfate; PSA; C18; GCB; MWCNTs	Micromass Quattro Premier XE (Waters Corporation, Milford, MA, USA)	0.003–0.034 μg/kg	55.3–118.7%	RSDs ≤ 20%	13	Not performed	Non compliant
[86]	Fish muscle	Switzerland	15	ACN with 1.5% AA and water	Disodium citrate sesquihydrate; sodium citrate; sodium chloride; magnesium sulfate; PSA	LCMS-8060NX (Shimadzu Corporation, Kyoto, Japan)	0.007–0.05 mg/kg	70–130%	Not reported	3	Not performed	Non compliant
[74]	Fish muscle, Poultry muscle, Swine muscle	USA	40	ACN with 1% AA and water	Disodium citrate sesquihydrate; sodium citrate; sodium chloride; magnesium sulfate	Agilent 6495D (Agilent Technologies, Santa Clara, CA, USA)	0.05–1.25 ng/g	72–151%	RSDs ≤ 20	31	Not performed	Explicitly claimed compliant
[82]	Oil	Poland	10	Acetonitrile (MeCN) + 0.15% formic acid (FA), water	sodium chloride; magnesium sulfate; ENVI-Carb	QTRAP 5500 (SCIEX, Framingham, MA, USA)	0.002–0.075 ng/g	72–104%	RSDs ≤ 20	2	Performed	Non compliant
[53]	Fish muscle	China	26	Water:ACN (2% *v*/*v* FA)	sodium chloride; magnesium sulfate; PSA; C18; Fe_3_O_4_-TiO	Shimadzu 8050 (Shimadzu Corporation, Kyoto, Japan)	0.025–0.050 μg/kg	71.3–116.3%	RSDs ≤ 20	2	Performed	Non compliant
[47]	Rice	Republic of Korea	35	Water:ACN (1:2), pH 2.4	sodium acetate; sodium chloride; magnesium sulfate; PSA; GCB	SCIEX Triple Quad 4500 (SCIEX, Framingham, MA, USA)	0.005–0.100 ng/g	86.5–126.4%	0.3–23.8%	21	Performed	Non compliant
[78]	Fish muscle	China	13	Acetonitrile (MeCN) + 0.10% HCl, MeOH	sodium chloride; PS-DVB	AB Sciex 4000 mass spectrometer (SCIEX, Framingham, MA, USA)	0.001–0.070 μg/kg	71.7–120%	RSDs ≤ 20	2	Performed	Apparently compliant
[48]	Fish muscle, Swine muscle, Cow muscle, Eggs, Liver, Milk	Italy	14	ACN, MeOH, Ammonium acetate, water	Disodium citrate sesquihydrate; sodium citrate; sodium chloride; magnesium sulfate; PSA; C18	API 6500 AB SCIEX (SCIEX, Framingham, MA, USA)	50–100 ng/kg	57–120%	RSDs ≤ 20	12	Not performed	Apparently compliant
[84]	Milk	UK (Scotland)	22	ACN with 0.1% FA	sodium acetate; Manetite-silica; Zirconiu dioxide; magnesium sulfate	Shimadzu 8050 (Shimadzu Corporation, Kyoto, Japan)	0.014–0.263 μg/kg	71.7–116%	RSDs ≤ 20	2	Performed	Apparently compliant
[72]	Fruits	Usa	10	Acetonitrile (MeCN) + Formic acid 1.5% *v*/*v*	sodium chloride; magnesium sulfa e; PSA; GCB	ABSciex 6500 QTRAP (SCIEX, Framingham, MA, USA)	0.2–5.6 ng/g.	60–115%	Not reported	3	Not performed	Non compliant
[71]	Fish muscle, Poultry muscle, Swine muscle, Cow muscle, Goat muscle, Crustacean, Eggs, Liver, Vegetables, Fruits, Milk, Rice	USA	16	Acetonitrile 0.15% FA, water	odium chloride; magnesium sulfate; PSA; UCT	ABScieX 6500 plus QTRAP (SCIEX, Framingham, MA, USA)	7–107 ppt	40–120%	Not reported	8	Performed	Non compliant
[70]	Fruits	Italy	9	Acetonitrile (MeCN) + 0.1% FA	sodium chloride; magnesium sulfate; GCB; ENVI-Carb	Sciex API 4000 (SCIEX, Framingham, MA, USA)	26–393 pg/g	75–97%	RSDs ≤ 20	9	Not performed	Non compliant
[15]	Fish muscle, Poultry muscle, Swine muscle, Crustacean, Eggs, Liver, Milk, Fruits, Vegetables	Belgium	25	ACN + NH_4_OH (1%)	Disodium citrate sesquihydrate; magnesium sulfate; PSA; ENVI-Carb	Q Exactive Focus™ Orbitrap (Thermo Fisher Scientific, Bremen, Germany)	0.002–1 μg/kg (not estimated for PFTrDS in tissue of animal origin, PFunDS in liver)	65–135%	RSD ≤ 25% with the exception of PFTrDS in animal-origin tissue and PFUnDS in liver	17	Not performed	Explicitly claimed compliant
[79]	Coffee	Republic of Korea	31	ACN + FA	sodium chloride; magnesium sulfate; PSA	Triple Quad 4500 system (SCIEX, Framingham, MA, USA)	0.012–0.260 ng/g	71.6–126.0%	RSD range 0.6 to 30%	19	Not performed	Non compliant
[76]	Milk	China	20	Water and ACN with 0.3% HCl	sodium chloride; PSA; C18; GCB	SCIEX Triple Quad 4500 (SCIEX, Framingham, MA, USA)	0.01–0.05 μg/L	72.8–111%	RSDs ≤ 20	9	Not performed	Apparently compliant
[75]	Fish muscle	China	30	ACN	sodium chloride; magnesium sulfate; PSA; C18; ENVI-Carb; Florisil	Agilent 6460 QQQ (Agilent Technologies, Santa Clara, CA, USA)	0.005–2.0 ng/g	64.5–128.0%	RSDs range 0.78–24.2%.	17	Performed	Apparently compliant
[85]	Fruits, Milk	UK (Scotland)	12	ACN	sodium chloride	QTRAP 5500 (SCIEX, Framingham, MA, USA)	0.25–0.1 ng/mL	80–120% with the exception of PFOS (69.3%)	RSD range 13.91–126.45%	4	Performed	Non compliant

## Data Availability

No new data were created or analyzed in this study. Data sharing is not applicable to this article.

## Supplementary Materials

The following supporting information can be downloaded at: https://www.mdpi.com/article/10.3390/foods15111872/s1, Table S1: PRIMA Abstract checklist; Table S2: Inclusion and exclusion criteria applied during the systematic literature screening following PRISMA guidelines; Table S3: RoB; Table S4: RoB Guidance and legend; Table S5: Methods; Table S6: PFAS Panels; Table S7: QA/QC methods; Table S8: Compliance with EU regulation.

## Abbreviations

The following abbreviations are used in this manuscript:

PFAS	Per- and polyfluoroalkyl substances
PFOS	Perfluorooctane sulfonic acid
PFOA	Perfluorooctanoic acid
EU POPs	European Union Reference Laboratory for Halogenated Persistent Organic Pollutants
IARC	International Agency for Research on Cancer
EFSA	European Food Safety Authority
PFNA	perfluorononanoic acid
PFHxS	perfluorohexane sulfonic acid
EURL	European Union Reference Laboratory
LC-MS/MS	Liquid chromatography coupled with tandem mass spectrometry
TOF	Time-of-flight
NTS	non-targeted screening
ACN	acetonitrile
dSPE	dispersive solid-phase extraction
QuEChERS	Quick, Easy, Cheap, Effective, Rugged, Safe
HRMS	high-resolution mass spectrometry
PRISMA	Preferred Reporting Items for Systematic Reviews and Meta-Analyses
PSA	primary–secondary amine
GCB	graphitized carbon black
QC	quality control
ESI	electrospray ionization
APCI	atmospheric pressure chemical ionization
QqQ	triple quadrupole
Q-TOF	quadrupole time of flight
RSD	relative standard deviation
LOQ	limit of quantification
FA	Formic acid
AA	Acetic acid
HCl	hydrochloric acid
PFBA	perfluorobutanoic acid
PFPrS	perfluoropropanesulfonic acid
N-MeFOSAA	N-methyl perfluorooctane sulfonamidoacetic acid
N-EtFOSAA	N-ethyl-perfluorooctane sulfonamidoacetic acid
MeOH	Methanol
KOH	potassium hydroxide
H_2_O	water
MTBE	methyl tert-butyl ether
QuEChERSER	Quick, Easy, Cheap, Effective, Rugged, Safe, Efficient, Robust
FDA	Food and Drug Administration
PES	polyether sulfone
PVDF	polyvinylidene fluoride
PP	polypropylene
NaCl	Sodium chloride
MgSO_4_	magnesium sulfate
FTS	fluorotelomer sulfonates
PSA	Primary secondary amine
PFCAs	Perfluoroalkyl carboxylic acids
PFSAs	Perfluorosulfonic acids
PFTeDA	Perfluorotetradecanoic acid
PFBS	Perfluorobutanesulfonic acid
PFDS	Perfluorodecanesulfonic acid
PS-DVB	styrene-divinylbenzene copolymer
C18	Octadecylsilane
MWCNTs	multi-walled carbon nanotubes
PEEK	polyether ether ketone
AOAC	Association of Official Analytical Collaboration
SMPR	Standard Method Performance Requirements
EPA	Environmental Protection Agency
ML	maximum level
TWI	Tolerable weekly intake
FOSA	Perfluorooctanesulfonamide
N-MeFOSE	N-methyl perfluorooctanesulfonamidoethanol
N-EtFOSE	N-ethyl perfluorooctanesulfonamidoethanol
ADONA	4,8-dioxa-3H-perfluorononanoic acid
NaDONA	Sodium 4,8-dioxa-3H-perfluorononanoate
PFPrA	perfluoropropanoic acid
PFAAs	perfluoroalkyl acids
WAX	weak anion exchange
2D-LC	Two-dimensional liquid chromatography
IM	Ion mobility
RoB	Risk of bias
ICV	Initial calibration verification
CCV	Continuing calibration verification
SSE	Signal suppression/enhancement
SRM	Standard reference material
RSD_R_	Intermediate precision
USDA	United States Department of Agriculture
KMD	Kendrick Mass Defect
EMR	Enhanced Matrix Removal
