# Supplementary material for: QuEChERS-Based LC-MS/MS and HRMS Methods for PFAS Determination in Food: A Systematic Review
Source: Foods. 2026 May 25;15(11):1872. doi: 10.3390/foods15111872 (PMC13257230; doi:10.3390/foods15111872)
Supplement: Supplementary file 1 [file foods-15-01872-s001.zip › Supplementary S1 and S2.pdf]

Supplementary Data

Analyses of per- and polyfluoroalkyl substances (PFAS) in food with QuEChERS-based analytical methods developed for LC-MS/MS and HRMS: a systematic review

Francesco Giuseppe Galluzzo[a], Gaetano Cammilleri[a], Licia Pantano[a], Vittorio Calabrese[b], Maria Drussilla Buscemi[a], Elisa Maria Domenica Messina[a]*, Calogero Alfano[a], Dario Bonomo[a], Andrea Pulvirenti[c], Andrea Macaluso[a], Vincenzo Ferrantelli[a], and Gianluigi Maria Lo Dico[a]

a Istituto Zooprofilattico Sperimentale della Sicilia "A. Mirri", 90129 Palermo, Italy

b Dipartimento di Scienze Biomediche e Biotecnologiche, Università degli studi di Catania, 95123 Catania, Italy

c Dipartimento Scienze della Vita, Università Degli Studi di Modena e Reggio Emilia, 41125 Modena, Italy

* Correspondence: elisa.messina@izssicilia.it

PRISMA 2020 flow diagram for new systematic reviews which included searches of databases and registers only

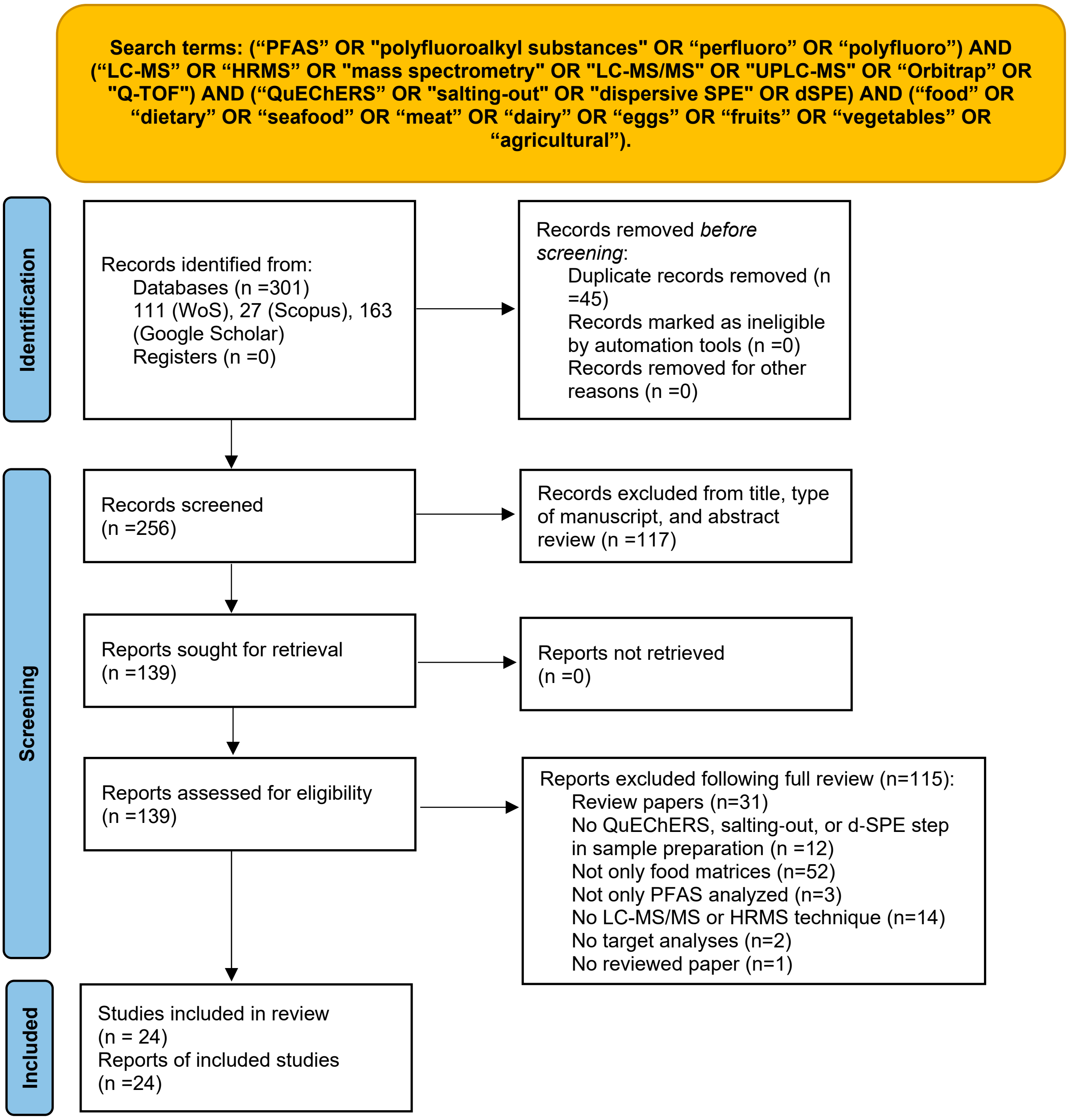

Source: Page MJ, et al. BMJ 2021;372:n71. doi: 10.1136/bmj.n71.

This work is licensed under CC BY 4.0. To view a copy of this license, visit https://creativecommons.org/licenses/by/4.0/

SI Figure S1. Selection and exclusion processes for the literature review.

PRISMA 2020 abstract checklist

Topic	No.	Item	Reported?
**TITLE**			
**Title**	1	Identify the report as a systematic review.	Yes
**BACKGROUND**			
**Objectives**	2	Provide an explicit statement of the main objective(s) or question(s) the review addresses.	Yes
**METHODS**			
**Eligibility criteria**	3	Specify the inclusion and exclusion criteria for the review.	Yes
**Information sources**	4	Specify the information sources (e.g. databases, registers) used to identify studies and the date when each was last searched.	Yes
**Risk of bias**	5	Specify the methods used to assess risk of bias in the included studies.	Yes
**Synthesis of results**	6	Specify the methods used to present and synthesize results.	Yes
**RESULTS**			
**Included studies**	7	Give the total number of included studies and participants and summarise relevant characteristics of studies.	Yes
**Synthesis of results**	8	Present results for main outcomes, preferably indicating the number of included studies and participants for each. If meta-analysis was done, report the summary estimate and confidence/credible interval. If comparing groups, indicate the direction of the effect (i.e. which group is favoured).	Yes
**DISCUSSION**			
**Limitations of evidence**	9	Provide a brief summary of the limitations of the evidence included in the review (e.g. study risk of bias, inconsistency and imprecision).	Yes
**Interpretation**	10	Provide a general interpretation of the results and important implications.	Yes
**OTHER**			
**Funding**	11	Specify the primary source of funding for the review.	Yes
**Registration**	12	Provide the register name and registration number.	Yes

Topic	No.	Item	Location where item is reported
**TITLE**			
**Title**	1	Identify the report as a systematic review.	1
**ABSTRACT**			
**Abstract**	2	See the PRISMA 2020 for Abstracts checklist	1
**INTRODUCTION**			
**Rationale**	3	Describe the rationale for the review in the context of existing knowledge.	2
**Objectives**	4	Provide an explicit statement of the objective(s) or question(s) the review addresses.	2
**METHODS**			
**Eligibility criteria**	5	Specify the inclusion and exclusion criteria for the review and how studies were grouped for the syntheses.	2-4, S1, S2, S3,
**Information sources**	6	Specify all databases, registers, websites, organisations, reference lists and other sources searched or consulted to identify studies. Specify the date when each source was last searched or consulted.	2-3
**Search strategy**	7	Present the full search strategies for all databases, registers and websites, including any filters and limits used.	2-3
**Selection process**	8	Specify the methods used to decide whether a study met the inclusion criteria of the review, including how many reviewers screened each record and each report retrieved, whether they worked independently, and if applicable, details of automation tools used in the process.	2-3
**Data collection process**	9	Specify the methods used to collect data from reports, including how many reviewers collected data from each report, whether they worked independently, any processes for obtaining or confirming data from study investigators, and if applicable, details of automation tools used in the process.	2-3

Topic	No.	Item	Location where item is reported
**Data items**	10a	List and define all outcomes for which data were sought. Specify whether all results that were compatible with each outcome domain in each study were sought (e.g. for all measures, time points, analyses), and if not, the methods used to decide which results to collect.	3
	10b	List and define all other variables for which data were sought (e.g. participant and intervention characteristics, funding sources). Describe any assumptions made about any missing or unclear information.	3-4
**Study risk of bias assessment**	11	Specify the methods used to assess risk of bias in the included studies, including details of the tool(s) used, how many reviewers assessed each study and whether they worked independently, and if applicable, details of automation tools used in the process.	3
**Effect measures**	12	Specify for each outcome the effect measure(s) (e.g. risk ratio, mean difference) used in the synthesis or presentation of results.	Not applicable. No clinical or exposure effect measures (e.g. risk ratios, mean differences) or quantitative meta-analyses were conducted; the review focuses on analytical performance parameters described narratively. Page 2-3.
**Synthesis methods**	13a	Describe the processes used to decide which studies were eligible for each synthesis (e.g. tabulating the study intervention characteristics and comparing against the planned groups for each synthesis (item 5)).	2-3
	13b	Describe any methods required to prepare the data for presentation or synthesis, such as handling of missing summary statistics, or data conversions.	4
	13c	Describe any methods used to tabulate or visually display results of individual studies and syntheses.	4
	13d	Describe any methods used to synthesize results and provide a rationale for the choice(s). If meta-analysis was performed, describe the model(s), method(s) to identify the presence and extent of statistical heterogeneity, and software package(s) used.	3-4

Topic	No.	Item	Location where item is reported
	13e	Describe any methods used to explore possible causes of heterogeneity among study results (e.g. subgroup analysis, meta-regression).	No subgroup/meta-regression; heterogeneity explored qualitatively in page 4-14.
	13f	Describe any sensitivity analyses conducted to assess robustness of the synthesized results.	No formal sensitivity analyses was conducted, results were narratively reported.
**Reporting bias assessment**	14	Describe any methods used to assess risk of bias due to missing results in a synthesis (arising from reporting biases).	3
**Certainty assessment**	15	Describe any methods used to assess certainty (or confidence) in the body of evidence for an outcome.	Confidence in the evidence base is discussed qualitatively in the Results and Discussions section (4-24)
**RESULTS**			
**Study selection**	16a	Describe the results of the search and selection process, from the number of records identified in the search to the number of studies included in the review, ideally using a flow diagram.	Figure S1, Figure 1
	16b	Cite studies that might appear to meet the inclusion criteria, but which were excluded, and explain why they were excluded.	Table S3 (reasons for exclusion at full-text screening)
**Study characteristics**	17	Cite each included study and present its characteristics.	4-24
**Risk of bias in studies**	18	Present assessments of risk of bias for each included study.	S4
**Results of individual studies**	19	For all outcomes, present, for each study: (a) summary statistics for each group (where appropriate) and (b) an effect estimate and its precision (e.g. confidence/credible interval), ideally using structured tables or plots.	S4, S5

Topic	No.	Item	Location where item is reported
**Results of syntheses**	20a	For each synthesis, briefly summarise the characteristics and risk of bias among contributing studies.	Characteristics and methodological limitations of the included methods are summarised narratively in the manuscript. No quantitative meta-analysis was performed.
	20b	Present results of all statistical syntheses conducted. If meta-analysis was done, present for each the summary estimate and its precision (e.g. confidence/credible interval) and measures of statistical heterogeneity. If comparing groups, describe the direction of the effect.	Not applicable. No statistical syntheses or meta-analyses were conducted; Supplementary Tables S4–S5 summarise individual study characteristics and performance parameters.
	20c	Present results of all investigations of possible causes of heterogeneity among study results.	24
	20d	Present results of all sensitivity analyses conducted to assess the robustness of the synthesized results.	5
**Reporting biases**	21	Present assessments of risk of bias due to missing results (arising from reporting biases) for each synthesis assessed.	5 and discussed qualitatively (5-24)
**Certainty of evidence**	22	Present assessments of certainty (or confidence) in the body of evidence for each outcome assessed.	Not formally assessed; discussed qualitatively (4-14)
**DISCUSSION**			
**Discussion**	23a	Provide a general interpretation of the results in the context of other evidence.	4-24
	23b	Discuss any limitations of the evidence included in the review.	24-25
	23c	Discuss any limitations of the review processes used.	24-25
	23d	Discuss implications of the results for practice, policy, and future research.	24-25
**OTHER INFORMATION**			

Topic	No.	Item	Location where item is reported
**Registration and protocol**	24a	Provide registration information for the review, including register name and registration number, or state that the review was not registered.	Not registered
	24b	Indicate where the review protocol can be accessed, or state that a protocol was not prepared.	17
	24c	Describe and explain any amendments to information provided at registration or in the protocol.	Not registered
**Support**	25	Describe sources of financial or non-financial support for the review, and the role of the funders or sponsors in the review.	Page 26
**Competing interests**	26	Declare any competing interests of review authors.	Page 26
**Availability of data, code and other materials**	27	Report which of the following are publicly available and where they can be found: template data collection forms; data extracted from included studies; data used for all analyses; analytic code; any other materials used in the review.	Page 26

Table S1 - PRISMA MAIN CHECKLIST [1]

Category	Inclusion criteria	Exclusion criteria
Publication type	Peer-reviewed articles, full research papers	Reviews, conference abstracts, books, posters, theses, non-peer-reviewed material
Time frame	Published between 2010 and 2025	Published before 2010 or after 2025
Matrix	Food and dietary exposure matrices (seafood, meat, dairy, eggs, fruits, vegetables, agricultural products)	Non-food, non-dietary, non-drinking water matrices (e.g., serum, environmental samples)
Analytes	Per- and polyfluoroalkyl substances (PFAS) with specific performance data	Multi-class methods without PFAS-specific performance data
Sample preparation	QuEChERS, salting-out, and/or d-SPE-based extraction/cleanup steps	Methods without any QuEChERS, salting-out, or d-SPE step
Analytical technique	LC-MS, LC-MS/MS, UPLC-MS, HRMS, Orbitrap, Q-TOF	Non-MS techniques only (e.g., GC-MS without PFAS suitability, non-chromatographic methods)
Method reporting	Sufficient methodological detail to assess extraction, cleanup, and validation	Insufficient methodological detail for evaluating extraction/cleanup or performance
Regulatory relevance	Data that usable to assess or discuss compliance with EU Reg. 2023/915 and/or 2022/1428	Studies with no relevance to food contaminant limits or analytical performance discussed in these regulations

Table S2 – Inclusion and exclusion criteria applied during the systematic literature screening following PRISMA guidelines.
